# From Carbohydrate to Biocompatible Carriers: Impact of Pegylation on the Physicochemical Properties and Quercetin Delivery Performance of Fructose Hydrothermal Carbons

**DOI:** 10.3390/polym18182189

**Published:** 2026-09-08

**Authors:** Ivan Bracanović, Ana Kalijadis, Lela Korićanac, Miljana Mirković, Mario Zlatović, Svetlana Butulija, Aleksandar Krstić

**Affiliations:** 1 Department of Materials, “Vinča” Institute of Nuclear Sciences-National Institute of the Republic of Serbia, University of Belgrade, Mike Petrovića Alasa 12-14, 11000 Belgrade, Serbia; ana.kalijadis@vin.bg.ac.rs (A.K.); miljanam@vin.bg.ac.rs (M.M.); svetlana8@vin.bg.ac.rs (S.B.); 2 Department of Molecular Biology and Endocrinology, “Vinča” Institute of Nuclear Sciences-National Institute of the Republic of Serbia, University of Belgrade, Mike Petrovića Alasa 12-14, 11000 Belgrade, Serbia; lela@vin.bg.ac.rs; 3 Faculty of Chemistry, University of Belgrade, Studentski trg 12-16, 11000 Belgrade, Serbia; mario@chem.bg.ac.rs; 4 Department of Physical Chemistry, “Vinča” Institute of Nuclear Sciences-National Institute of the Republic of Serbia, University of Belgrade, Mike Petrovića Alasa 12-14, 11000 Belgrade, Serbia

**Keywords:** hydrothermal carbonization, fructose, functionalization, cytotoxicity, quercetin, drug carrier

## Abstract

The aim of this study was to investigate the effect of PEG functionalization of hydrothermal carbon (HTC) on quercetin adsorption and desorption kinetics and, through that, evaluate the potential of functionalized HTC as a carrier for quercetin. Hydrothermal carbon (HTC) was synthesized using fructose as a precursor at a temperature of 160 °C. Structural and morphological analyses using X-ray diffraction and scanning electron microscopy (SEM) confirmed an amorphous carbon structure and microspherical particles with an average size of 5.6 µm. X-ray photoelectron spectroscopy (XPS) and Fourier Transform Infrared (FT-IR) spectroscopy characterization revealed a surface enriched with hydroxyl and carboxyl groups, which facilitated successful PEG modification. Surface modification was further corroborated by a zeta potential shift from –26.4 mV to –16.4 mV. Cytotoxicity assays in *MRC-5* and *HeLa* cell lines confirmed high biocompatibility, with cell viability remaining above 70%. Quercetin binding experiments showed that PEG functionalization increased binding capacity up to 14%, reaching 19.50 mg/g for PEG-functionalized fructose-derived carbon. Desorption kinetics followed a pseudo-second-order model, with the PEG-modified sample exhibiting significantly slower rates than the unmodified sample. These findings indicate that PEG functionalization can improve the adsorption/desorption properties of HTC compared with the pristine material, highlighting its potential as a promising, environmentally friendly, and efficient delivery system for quercetin.

## 1. Introduction

Hydrothermal carbonization (HTC) is a highly efficient method for synthesizing carbon materials that reduces energy consumption and eliminates the use of oil or fossil-derived precursors, making the process more cost-effective and aligned with green chemistry principles [1,2]. This thermochemical process occurs at relatively low temperatures and uses self-generated pressure in an aqueous environment to convert various carbohydrates and organic waste into carbon-based materials [3,4]. Unlike traditional pyrolysis or carbonization, which requires high temperatures and dry feedstock, HTC allows direct transformation of “wet” biomass, significantly reducing the carbon footprint of the synthesis. The surface properties of the resulting material mainly depend on the choice of precursor and its concentration, as well as the temperature and process duration. Previous research indicates that carbons produced through this method are adjustable for many uses, including energy and gas storage, removal of heavy metals and organic pollutants, and potential antibacterial and cancer therapies [5,6].

Hydrothermal carbons (HTC_s_) distinguish themselves from other carbonaceous materials by inherently developing oxygen-rich functional groups during the carbonization process [7,8,9]. This spontaneous introduction of functionalities, such as carboxyl, anhydride, ketone, lactone, ether, phenol, and alcohol groups, excludes the necessity for additional chemical treatments and greatly simplifies subsequent surface design [10]. Such intrinsic chemical diversity is particularly advantageous in biomedical contexts, where surface tailoring is critical to enhance biocompatibility, optimize drug-loading efficiency, and mitigate cytotoxic effects [5]. HTC-derived carbons could serve as a highly adaptable scaffold, enabling both electrostatic interactions and covalent conjugation with a broad spectrum of therapeutic agents and antioxidants. This versatility not only facilitates controlled drug delivery but also opens pathways for multifunctional carriers capable of synergistic therapeutic action, thereby positioning HTC-derived carbons as a promising candidate for advanced biomedical applications.

Quercetin, chemically known as 3,3′,4′,5,7-pentahydroxyflavone, is a naturally occurring flavonoid widely distributed in various plant-based foods, including fruits, vegetables, seeds, and grains, with particularly high concentrations found in onions, apples, and berries [11,12]. As one of the most studied polyphenolic compounds, quercetin has attracted significant attention for its diverse biological activities, including antioxidant, anti-inflammatory, anticarcinogenic, antiviral, and cardioprotective effects [11]. Quercetin’s therapeutic potential lies in its ability to modulate various signaling pathways and mitigate oxidative-stress-induced damage. These properties make quercetin a promising candidate for preventing and treating numerous chronic diseases, including cardiovascular disorders, cancer, diabetes, and neurodegenerative diseases [11,12]. Quercetin’s antioxidant potential primarily stems from its ability to scavenge reactive oxygen species (ROS), chelate metal ions, and regulate cellular antioxidant pathways, thereby mitigating oxidative-stress-induced damage [11,12,13]. Additionally, its anti-inflammatory properties are associated with modulating key inflammatory mediators, including cytokines and enzymes, positioning quercetin as a potential therapeutic agent for inflammation-driven diseases. Furthermore, its anticancer activity is linked to mechanisms such as apoptosis induction, cell cycle arrest, and suppression of angiogenesis and metastasis, making it an attractive candidate for cancer therapy [13]. Beyond these effects, quercetin also demonstrates antiulcer, antimicrobial, antidiabetic, and immunomodulatory activities, highlighting its broad therapeutic potential [13]. Despite its promising biological profile, quercetin’s clinical potential remains limited because of its poor bioavailability, low aqueous solubility, rapid metabolism, and short half-life [14]. These pharmacokinetic challenges highlight the need for innovative strategies to improve bioavailability and minimize potential adverse effects [14]. Exploring drug delivery systems such as nanoparticle encapsulation, liposomes, and polymeric carriers demonstrates promising progress in overcoming limitations, fostering optimism about future advancements in quercetin delivery. For this purpose, researchers have explored various carbon materials as quercetin carriers, including functionalized graphene oxide, graphene quantum dots, and carbon nanotubes [15,16,17].

Although many carbon-based materials have remarkable electrical, mechanical, and optical properties, cytotoxicity, aggregation, and poor dispersibility in physiological environments can hinder their direct biomedical use [18,19,20]. Polyethylene glycol (PEG) functionalization addresses these limitations by improving hydrophilicity, reducing nonspecific protein adsorption, and minimizing immune recognition, which prolongs circulation time in vivo [21,22]. The PEG coating forms a steric barrier around materials, preventing aggregation and reducing cellular stress responses [22,23]. As a result, PEG-modified carbon-based nanomaterials demonstrate enhanced biocompatibility, making them safer and more effective for applications such as drug delivery. However, these carbon materials often come with high commercial costs or involve environmentally harmful synthesis methods, especially when compared to carbon derived from hydrothermal carbonization.

Our previous work examined the potential use of two types of hydrothermal carbon materials synthesized from different precursors as quercetin carriers, compared with multiwalled nanotubes, which have been shown to be successful drug carriers [3]. By comparison, the current research provides a more detailed analysis of the role of PEG functionalization of fructose-derived HTC in quercetin adsorption/desorption kinetics for a drug delivery system. By exploiting the oxygen-containing groups generated during synthesis and further tailoring the surface through PEG functionalization, we aimed to investigate PEG’s role in potentially enhancing drug–material interactions, providing a foundation for the rational design of thermally tuned, biocompatible antioxidant delivery systems.

## 2. Materials and Methods

### 2.1. Materials

D-(−)-Fructose (purity ≥ 99%), polyethylene glycol (PEG) (purity ≥ 99%, average mol wt 8000), and quercetin (purity ≥ 95%) were purchased from Sigma-Aldrich (Burlington, MA, USA). Tetrahydrofuran (THF) (purity ≥ 99.9%) and Pyridine (p.a., ACS Reagent) were purchased from VWR International (Radnor, PA, USA). Thionyl chloride (purity > 99.5%) was obtained from Acros Organics (Antwerpen, Geel, Belgium).

### 2.2. Synthesis and Modification

HTC-derived carbon was obtained by preparing a 3 M fructose solution and transferring 28 mL of it into a Teflon-lined reactor. The sealed autoclave was maintained at 160 °C for 6 h, after which the solid hydrothermal product (HTC_f_) was collected. The material was repeatedly washed with distilled water and subsequently with ethanol until the suspension became completely colorless, then dried overnight at 60 °C [3].

Surface functionalization with polyethylene glycol (PEG) was carried out through an acyl-chloride activation step. To introduce acyl chloride groups, 600 mg of the carbon powder was dispersed in a mixture of SOCl_2_ (75 mL) and THF (40 mL) and refluxed at 80 °C for 36 h. The solid was isolated by filtration, rinsed with THF, and dried under ambient conditions. The activated carbon was then suspended in THF (25 mL) and pyridine (45 mL), after which 1.1 g of PEG was added. The reaction mixture was stirred for 24 h at room temperature. The PEG-modified carbon (HTC_f-PEG_) was obtained by filtration, washing with THF, and drying at room temperature [3].

### 2.3. Scanning Electron Microscopy (SEM)

Surface morphology was evaluated using FESEM, SCIOS 2 Dual Beam, Thermo Fisher Scientific, (Waltham, MA, USA), field emission scanning electron microscopy. Particle size measurements were obtained by analyzing the acquired SEM micrographs with ImageJ (version 1.46r), following standard image-processing and thresholding procedures.

### 2.4. X-Ray Diffraction (XRD)

Structural characteristics of the HTC were examined by X-ray diffraction (XRD) using a Rigaku Ultima IV diffractometer (Rigaku Corporation, Tokyo, Japan) equipped with Cu Kα radiation. Measurements were performed at 40 kV and 40 mA over the 2*θ* range of 10–90°. Data were collected in continuous-scan mode with a step size of 0.02° and a scan rate of 5°/min using a D/TeX Ultra high-speed detector. The HTC sample was mounted on a monocrystalline Si holder (Shuyun Instrument, Shanghai, China) before analysis. Result was processed with the PDXL2 software (Version: 2.8.4.0, database version: 2.2301).

### 2.5. FT-IR Spectroscopy

Fourier Transform Infrared spectroscopy (FT-IR) was used to identify surface functional groups and assess the chemical composition of the HTC materials. Spectra were collected using a Thermo Fisher Scientific Nicolet iS5 spectrometer (Thermo Fisher Scientific Inc., Wilmington, MA, USA) and processed with the Omnic 9 software package (version 9.7.46). Samples were prepared by thoroughly mixing the material with KBr and pressing the mixture into KBr pellets.

### 2.6. X-Ray Photoelectron Spectroscopy (XPS)

X-ray photoelectron spectroscopy (XPS) measurements were performed using a SPECS system equipped with an XP50M X-ray source for the Focus 500 and a PHOIBOS 100 analyzer (all from SPECS Surface Nano Analysis GmbH, Berlin, Germany). A monochromatic Al Kα radiation source (1486.74 eV) operated at 12.5 kV and 16 mA served as the excitation beam. Samples were mounted on adhesive copper foil to ensure firm mechanical support and reliable electrical grounding. Survey spectra spanning 0–1000 eV (binding energy) were collected at a constant pass energy of 40 eV, with a step size of 0.5 eV and a dwell time of 0.2 s. High-resolution scans of individual core levels were acquired using a pass energy of 20 eV, an energy increment of 0.1 eV, and a dwell time of 2 s. Data acquisition was carried out with SpecsLab software (Version: 1.6), while spectral processing and peak fitting were performed in CasaXPS (Version: 2.3.16Dev52). All spectra were corrected using a standard Shirley background.

### 2.7. Zeta Potential

The zeta potential was measured using a Malvern Zetasizer Nano Series (nano-ZS, Malvern Panalytical, Great Malvern, UK), and the data were processed using Zetasizer software (version 6.32).

### 2.8. Cell Culture and Treatment

*MRC-5* (*CCL-171*) human embryonal lung fibroblasts and *HeLa* (*CCL-2*) human cervical carcinoma cells were sourced from ATCC (Rockville, MD, USA). Both cell lines were maintained in Dulbecco’s Modified Eagle’s Medium (Capricorn Scientific GmbH, Ebsdorfergrund-Dreihausen, Germany) supplemented with 10% fetal bovine serum (ThermoFisher Scientific Inc., Waltham, MA, USA) and a penicillin (10,000 U/mL)/streptomycin (10 mg/mL) solution (Capricorn Scientific GmbH). Cultures were incubated under standard conditions, in a humidified atmosphere containing 5% CO_2_ at 37 °C (ESCO, Lifesciences Group, Singapore, Singapore).

### 2.9. Cytotoxicity Assay

*MRC-5* and *HeLa* cells were seeded at a density of 2000 cells per well in flat-bottomed 96-well microtiter plates (Sarstedt, Numbrecht, Germany) and incubated overnight. Stock material solutions were prepared in sterile distilled H_2_O at a concentration of 2 mM. Before the experiment, working concentrations (50, 100, and 200 µg/mL) of non-modified fructose-derived carbon (HTC_f_), a functionalized sample (HTC_f_-_PEG_), and HTC_f_ and HTC_f-PEG_ samples with bonded quercetin (HTC_f_-Q, HTC_f-PEG_-Q) were prepared in cell culture medium, and cells were exposed for either 48 or 72 h. Cell viability was assessed using the sulforhodamine B (SRB) assay as previously described [24]. Cell monolayers were fixed with 10% trichloroacetic acid (Sigma-Aldrich Chemie GmbH, Schnelldorf, Germany) for 1 h at 4 °C, then washed with distilled water. Cells were stained with 0.4% SRB (Sigma-Aldrich Chemie GmbH) in 1% acetic acid (Centrohem, Stara Pazova, Serbia), and excess stain was removed by washing with 1% acetic acid. Protein-bound dye was solubilized in 10 mM Tris base (Sigma-Aldrich Chemie GmbH). Absorbance was measured at 550 nm with a reference wavelength of 690 nm using a microplate reader (Wallac VICTOR2 1420 Multilabel counter, PerkinElmer, Turku, Finland).

### 2.10. Statistical Analysis

All measurements were performed in quadruplicate, and each experiment was conducted twice. Group differences were evaluated using an independent-samples *t*-test (*p* < 0.05). Results are reported as mean ± standard deviation (S.D.).

### 2.11. Adsorption Experiment–Quercetin Binding

Quercetin binding to the HTC_f_ was carried out using the batch adsorption kinetics method. A mass of 20 mg of HTC_f_ and HTC_f_-_PEG_ samples was immersed in a white plastic tube containing 60 mL of quercetin solution with an initial concentration of 10 ppm, and the mixture was shaken for 24 h, in the dark, at 150 rpm at room temperature (25 °C) [3,4]. Quercetin was dissolved in a water–ethanol solution with a 4:1 volume ratio, and the pH of the prepared solution was 6.8. After the defined time, the equilibrium concentration of quercetin was measured using an LLG-uni SPEC 2 Spectrophotometer (LLG Labware, Meckenheim, Germany) at 370 nm. Before analysis, samples were properly diluted with deionized water. Concentration of quercetin was calculated from a calibration curve. The concentration of bonded quercetin was calculated by the following equations [4]:

(1) $$C_{sol}=\frac{C_{0}{\times A}_{sol}}{A_{0}}$$

Here, C_sol_ is the concentration of quercetin in the solution after adsorption in mg/L, C_0_ is the initial concentration of quercetin in mg/L, A_0_ is the absorption value at wavelength 370 nm, and A_sol_ is the absorption value of the solution after adsorption at wavelength 370 nm.

(2) $$m_{ads}=\frac{\left(C_{0}-C_{sol}\right)}{m_{mat}}\times V$$

Here, m_ads_ is the amount of quercetin adsorbed onto 1 g of carbon material in mg, m_mat_ is the amount of HTC material in g, and V is the volume of solution used for the adsorption experiment in l.

(3) $$Q(\%)=\frac{\left(C_{0}-C_{sol}\right)}{C_{0}}\times 100$$

Here, Q (%) is the percentage of quercetin adsorbed onto the HTC material.

For describing the kinetic behavior of quercetin, experimental data were fitted to non-linear equations of pseudo-first-order (Equation (4)) and pseudo-second-order kinetics (Equation (5)) models [4]:

(4) $$q_{t}=q_{e}\times (1-e^{-k_{1}\times t})$$

(5) $$q_{t}=q_{e}\times {\left(\frac{1}{q_{e}}+k_{2}+t\right)}^{-1}$$

where q_t_ and q_e_ (mg/g) are the amounts of quercetin desorbed at the time t (min) at equilibrium, respectively, and k_1_ (min^−1^) and k_2_ (g mg^−1^ min^−1^) are the pseudo-first- and pseudo-second-order rate constants [4].

### 2.12. Desorption Experiment

The desorption experiment was performed using 20 mg of quercetin-adsorbed samples in a 60 mL mixture of ethanol and water (1:4) for 48 h, with aliquots taken at defined time intervals (15 min, 30 min, 1 h, 2 h, 4 h, 24 h, and 48 h). After the defined time, the equilibrium concentration of quercetin was measured using an LLG-uni SPEC 2 Spectrophotometer (LLG Labware, Meckenheim, Germany) at 370 nm. The percentage of quercetin in the solution was calculated using Formulas (1) and (3) [25].

## 3. Results and Discussion

### 3.1. SEM

**Figure 1:**
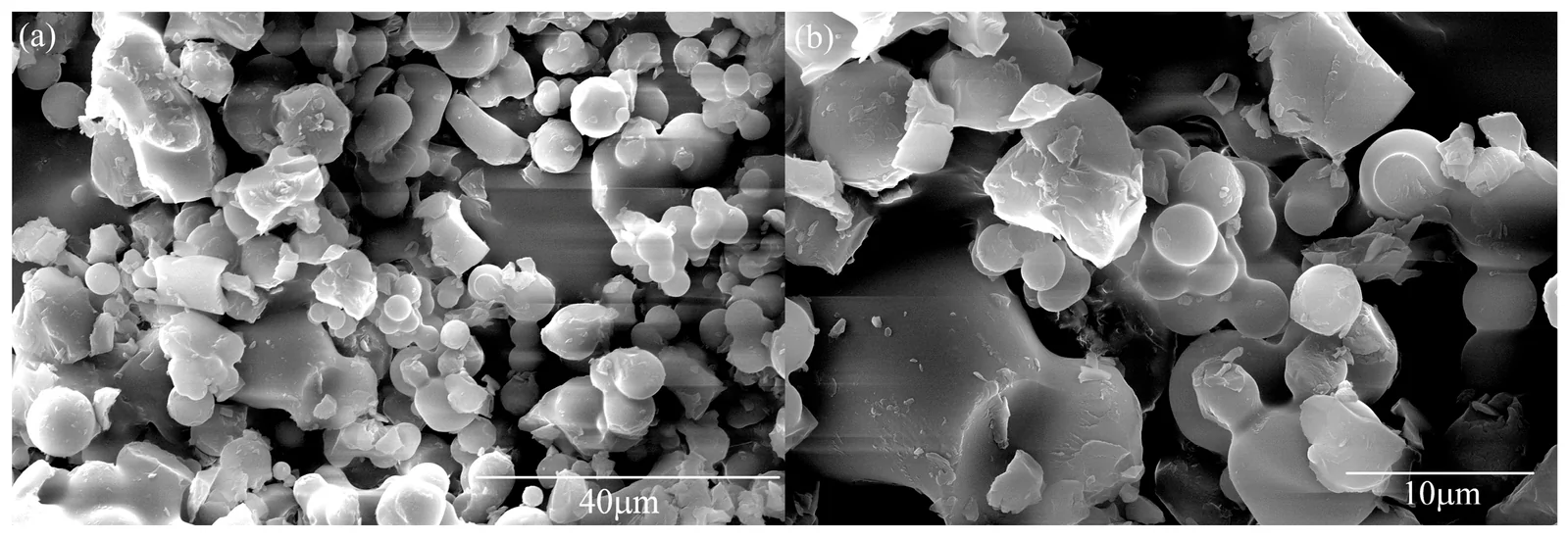
SEM images of HTC_f_ at magnifications of (**a**) 2000× and (**b**) 5000×.

Figure 1 presents SEM pictures of the HTC_f_ at two different magnifications. A sphere-size discrepancy and irregularly shaped agglomerates are noticeable, with sizes ranging from 2 µm to 17 µm. Particle size analysis shows that HTC_f_ particles are predominantly microdimensional, with an average particle size of 5.6 µm. Agglomeration and microdimensional particles likely originate from fructose’s low activation energy, which leads to particle enlargement during hydrothermal carbonization [26]. Fructose, being a keto-hexose, is already in the structural form required for rapid dehydration, so it converts into 5-hydroxymethylfurfural and related intermediates with comparatively low energetic demand [27].

### 3.2. XRD Analysis

**Figure 2:**
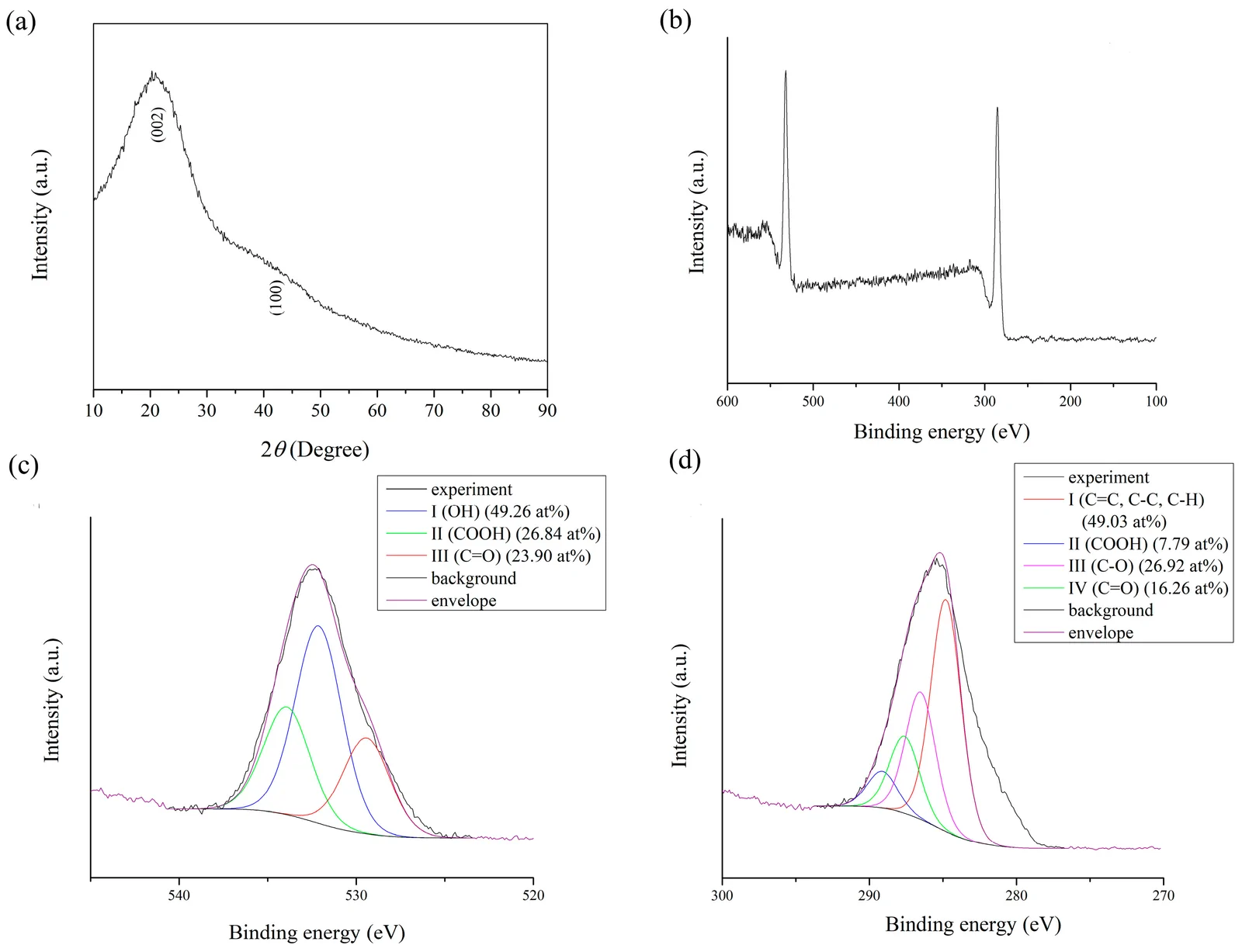
(**a**) XRD, (**b**) XPS, (**c**) XPS-O1s, and (**d**) XPS-C1s for HTC_f_ sample.

Figure 2a illustrates the X-ray diffractogram of HTC_f_. The diffraction profile shows two distinct peaks, corresponding to the (002) and (100) plane reflections of an amorphous carbon structure [3,28].

### 3.3. XPS Analysis

Figure 2b shows the survey spectra of HTC_f_ taken in the binding energy range from 200 eV to 600 eV. Peaks at approximately 285 eV and 534 eV correspond to C1s and O1s, respectively. Figure 2c shows O1s spectra that offer insight into the distribution of oxygen-based surface functional groups, showing that of the 28.46 at% of oxygen, 49.26 at% are hydroxyl functional groups (532 eV), 26.84 at% are carboxyl functional groups (534.5 eV), and 23.9 at% are carbonyl groups (529.7 eV). Figure 2d shows the C1s spectra and indicates that 49.03 at% are sp^2−^ and sp^3−^ carbon atoms [3,29,30]. Deconvolution tables with detected XPS peaks are given in Table 1.

### 3.4. FT-IR Spectroscopy

**Figure 3:**
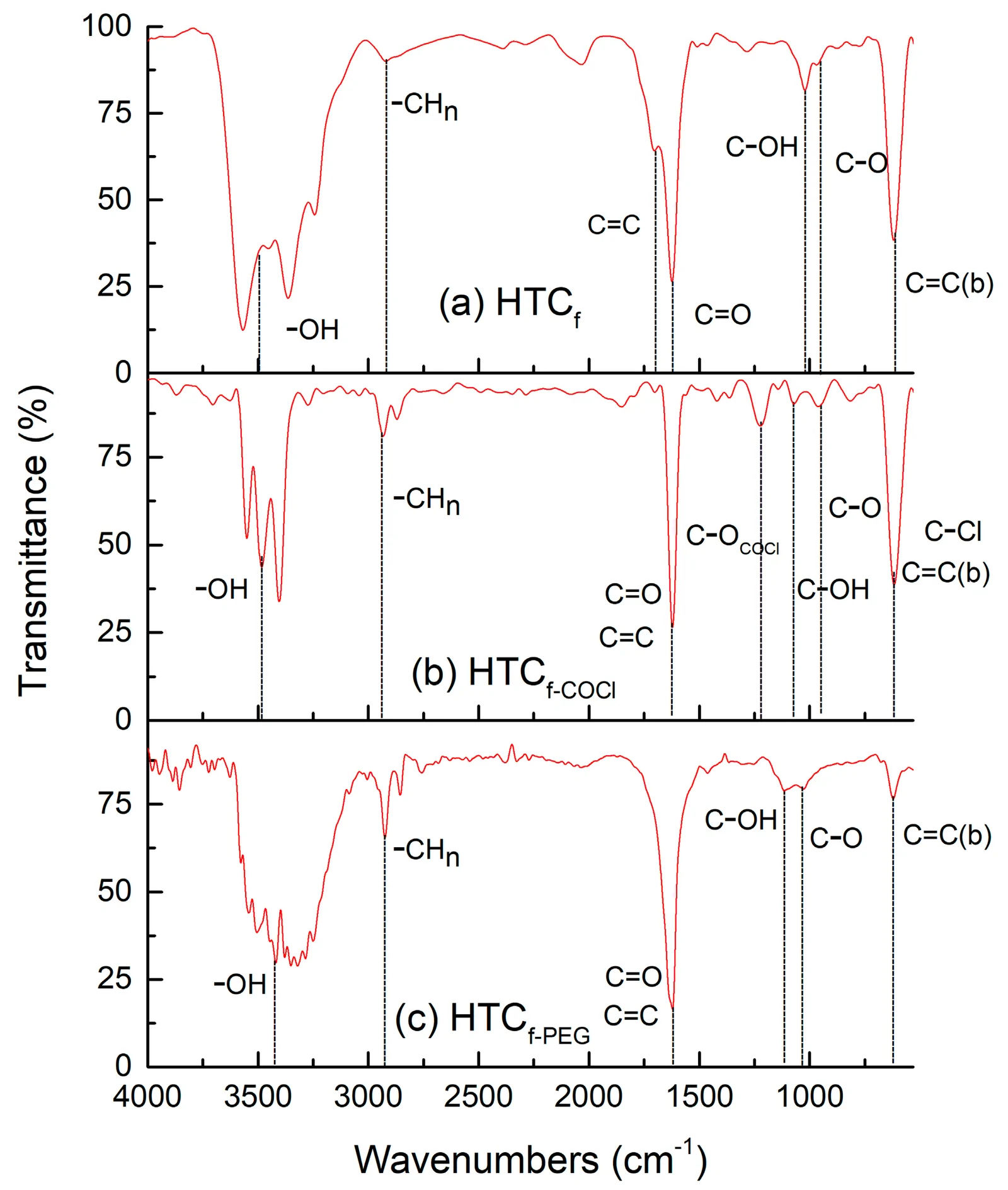
FT-IR spectra of (**a**) HTC_f_, (**b**) HTC_f-COCl_ and (**c**) HTC_f-PEG_ samples.

Figure 3 shows the FT-IR spectra of three synthesized and modified HTC_f_ samples. All examined samples show a broad peak between 3700 cm^−1^ and 3100 cm^−1^, attributed to the O-H stretching vibration of hydroxyl groups. The region between 3000 cm^−1^ and 2800 cm^−1^ is attributed to C-H stretching of sp^2−^ and sp^3−^ hybridized carbon [3,22]. The peak between 1700 cm^−1^ and 1600 cm^−1^ can be assigned to C-O stretching vibrations of carboxyl functional groups and C=C stretching vibrations. For the C=C stretching vibration in HTC_f-COCl_ (Figure 3b) and HTC_f-PEG_ (Figure 3c), the C=C is covered by a C-O stretching vibration, which can be confirmed by the C=C bending vibration at around 622 cm^−1^ for all samples [3,31]. The main difference between these spectra is in the 1250–622 cm^−1^ range, indicating successful functionalization. In this range, for HTC_f_ (Figure 3a), the peaks at 1010 cm^−1^ and 950 cm^−1^ correspond to C-OH and C-O stretching vibrations, respectively, confirming the presence of oxygen functional groups on the surface, namely hydroxyl (-OH) and carboxyl (-COOH) groups [3,23]. The spectra for HTC_f-COCl_ (Figure 3b) show a shift in a peak that refers to the C-OH vibration at 1070 cm^−1^, and a new peak at 1240 cm^−1^ for C-O vibration, which is unique for acyl chloride functional groups. The peak at 622 cm^−1^ is also more intense, which can be attributed to C=C and C-Cl stretching vibrations. These observations confirmed successful conversion of acidic functional groups to acyl chlorides [3,31]. The spectrum for HTC_f-PEG_ (Figure 3c) shows a shift in peaks that refer to the C-OH and C-O stretching vibrations between 1250 cm^−1^ and 1000 cm^−1^, and the absence of a peak at 1240 cm^−1^, which indicates a successful PEG modification of HTC_f_ into HTC_f-PEG_ [3,32].

### 3.5. Zeta Potential

Table 2 summarizes the zeta potential measurements for both HTC_f_ and its PEG-modified counterpart, HTC_f-PEG_. The variation in surface potential following PEGylation provides clear evidence of successful surface modification. Specifically, PEG functionalization shifts the zeta potential toward more positive values, indicating a reduction in overall negative surface charge. As previously demonstrated in an earlier study, this change can be attributed to the shielding effect and steric stabilization imparted by the PEG chains [3,31].

### 3.6. Cytotoxic Activity Evaluation

**Figure 4:**
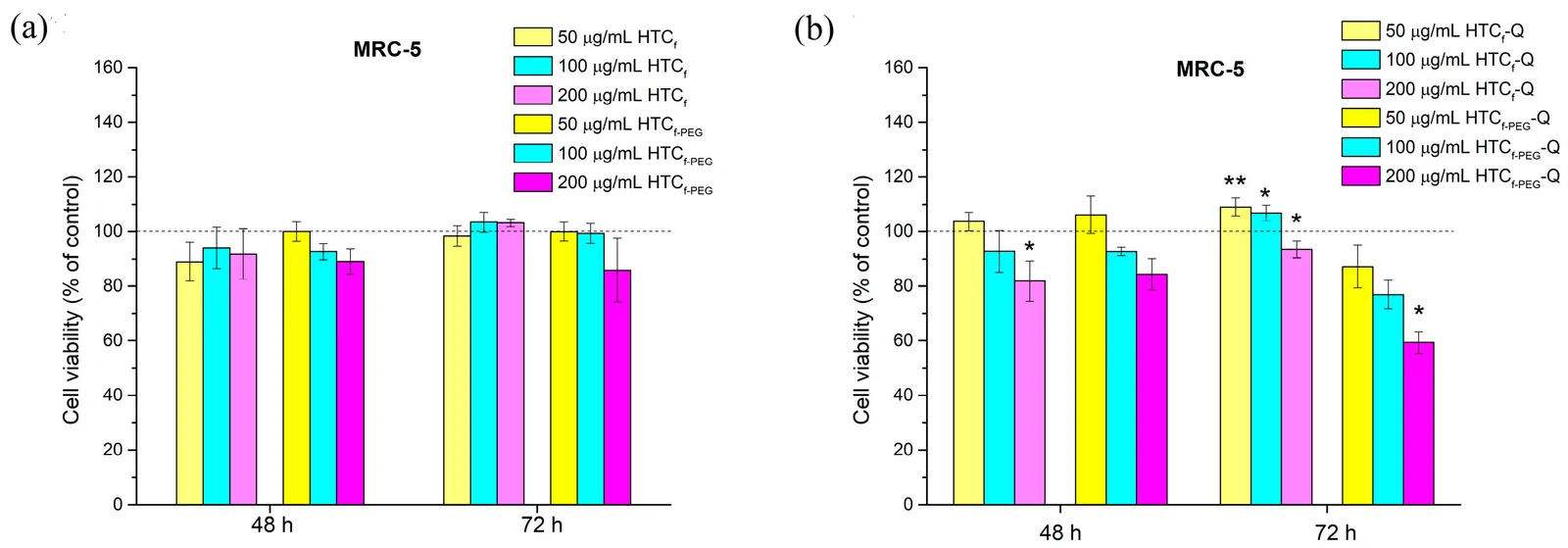
The cytotoxic effects of HTC_f_ and HTC_f_-_PEG_ (**a**) and HTC_f_-Q and HTC_f-PEG_-Q (**b**) HTC materials on non-malignant *MRC-5* human embryonic fibroblast cells, assessed 48 and 72 h after treatment, using the SRB method. Mean values were obtained from two independent experiments conducted in quadruplicate and are presented as a percentage of the control ± SD. The control value was set at 100 percent. Statistical significance—* *p* < 0.05; ** *p* < 0.01.

**Figure 5:**
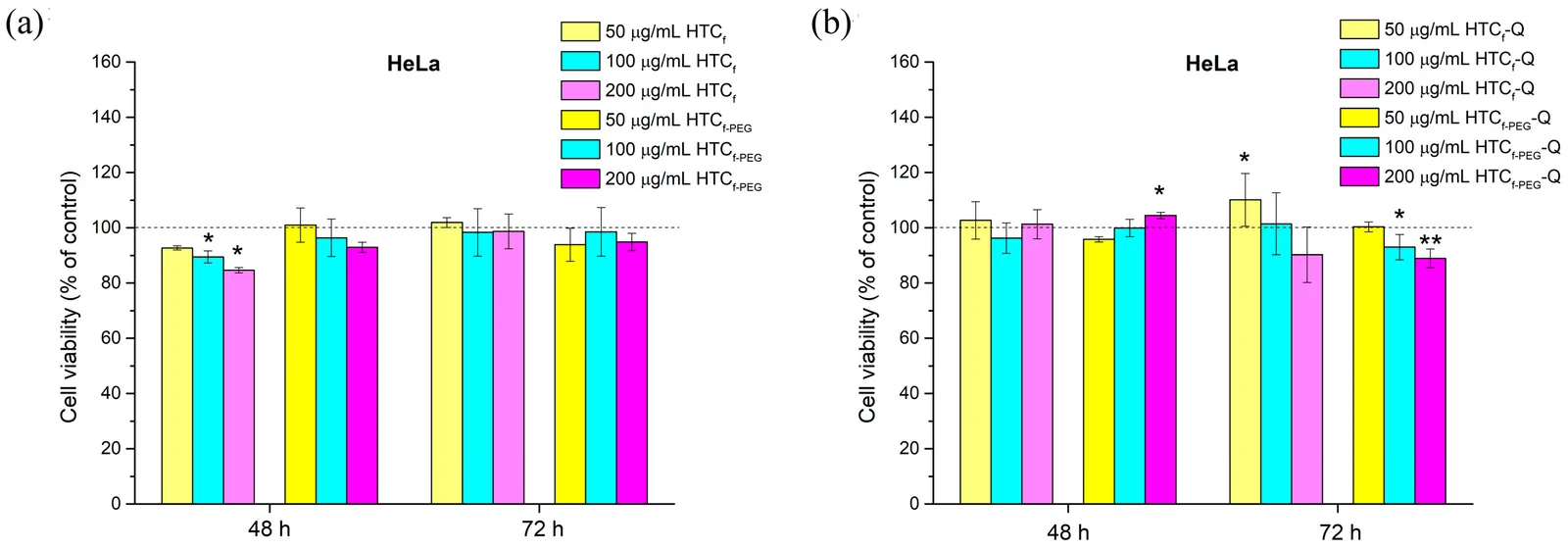
The cytotoxic effects of HTC_f_ and HTC_f-PEG_ (**a**) and HTC_f-PEG_-Q and HTC_f-PEG_-Q (**b**) nanomaterials on *HeLa* human cervical carcinoma cells, assessed 48 and 72 h after treatment, using the SRB method. Mean values were obtained from two independent experiments performed in quadruplicate and are presented as a percentage of the control ± SD. The control value was set at 100 percent. Statistical significance—* *p* < 0.05; ** *p* < 0.01.

The chemosensitivity of the synthesized HTC materials was evaluated on *MRC-5* and *HeLa* cells. The evaluation was time- and dose-dependent (Figure 4 and Figure 5). No significant cytotoxicity was detected in human fibroblasts following treatment with HTC_f_ or HTC_f-PEG_ at the analyzed time points (Figure 4a). Under identical experimental conditions, HTC_f_-Q reduced cell viability at 48 and 72 h post-treatment when the highest concentration (200 µg/mL) was administered (*p* < 0.05). In contrast, lower concentrations (50 and 100 µg/mL) at 72 h post-treatment promoted *MRC-5* cell growth. HTC_f-PEG_-Q reduced the viability of *MRC-5* cells only at 72 h post-treatment with the highest concentration (*p* < 0.05) (Figure 4b).

The HTC_f_ sample decreased *HeLa* cell viability by 89% and 84% at 48 h after treatment with 100 and 200 µg/mL, respectively (*p* < 0.05). However, cell viability fully recovered after 72 h, reaching the untreated control level. HTC_f-PEG_ was not cytotoxic on *HeLa* cells (Figure 5a). HTC_f_-Qdemonstrated high biocompatibility with *HeLa* cells.

The lowest concentration of HTC_f_-Q slightly increased *HeLa* cell viability at 72 h (*p* < 0.05). HTC_f_-_PEG_-Q initially slightly stimulated *HeLa* cell growth 48 h after treatment at 200 µg/mL (*p* < 0.05), whereas prolonging the treatment period decreased viability at 100 and 200 µg/mL (*p* < 0.05, *p* < 0.01) (Figure 5b).

These results indicate high biocompatibility of these HTC materials. According to the literature, materials are considered cytocompatible if the percentage of viable cells is at least 70% [33,34]. Therefore, all tested materials meet the criteria for biocompatibility, despite the statistically significant changes observed with the application of 200 μg/mL HTC_f-PEG_-Q material.

### 3.7. Quercetin Binding on Synthesized Materials

**Figure 6:**
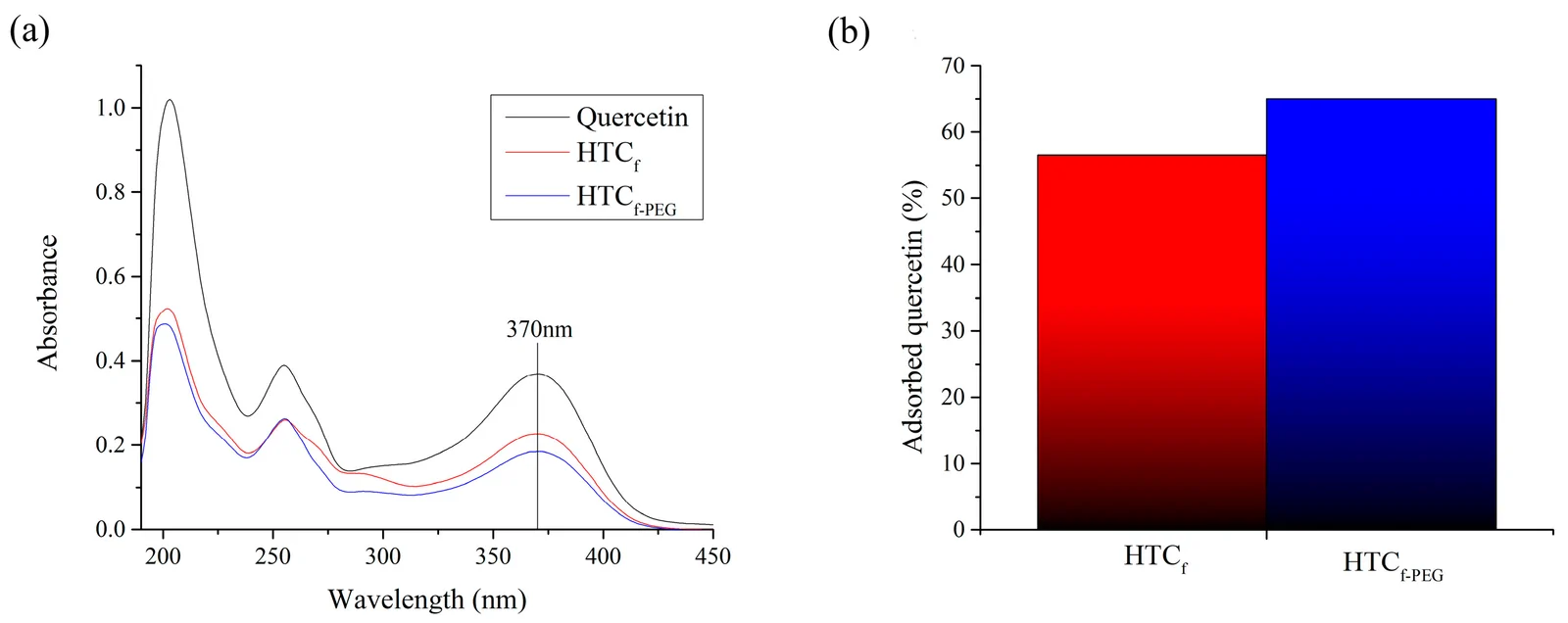
(**a**) UV/VIS spectra of 10 ppm quercetin solution and of HTC_f_ and HTC_f-PEG_ samples in quercetin solution after shaking for 24 h and (**b**) percentage of bonded quercetin on fructose HTCs.

Quercetin binding experiments were performed to investigate the role of PEG in quercetin binding on fructose-derived hydrothermal carbon. Figure 6a shows the UV/VIS spectra of pure quercetin solution at 10 ppm, and of HTC_f_, and HTC_f-PEG_ samples after 24 h of shaking in quercetin solution. The characteristic quercetin peak appears at 370 nm, and the peak height decreases significantly between the pure quercetin solution and the sample-containing solutions, confirming successful quercetin binding to the material surface. Additionally, the quercetin peak height differs notably between HTC_f_ samples before and after PEG functionalization, indicating enhanced binding. The amounts of bonded quercetin on HTC_f_ and HTC_f-PEG_ samples, calculated by Equation (2), were 16.95 mg/g and 19.50 mg/g, respectively. Percentages of attached quercetin in the tested samples are shown in Figure 6b. The data indicate that PEG functionalization of fructose-derived HTC materials enhances quercetin binding capacity and demonstrates its potential to improve quercetin carrier potential. It can be assumed that PEG, being a highly hydrophilic polymer, acts as a molecular mediator when introduced into a solution containing quercetin, a compound with inherently poor aqueous solubility [23]. Under this assumption, PEG can form hydrogen bonds with quercetin, increasing its solubility and reducing its tendency to crystallize or aggregate. In this stabilized state, quercetin could become more available for adsorption onto the surface of carbon materials [23].

### 3.8. Quercetin Desorption Kinetics

**Figure 7:**
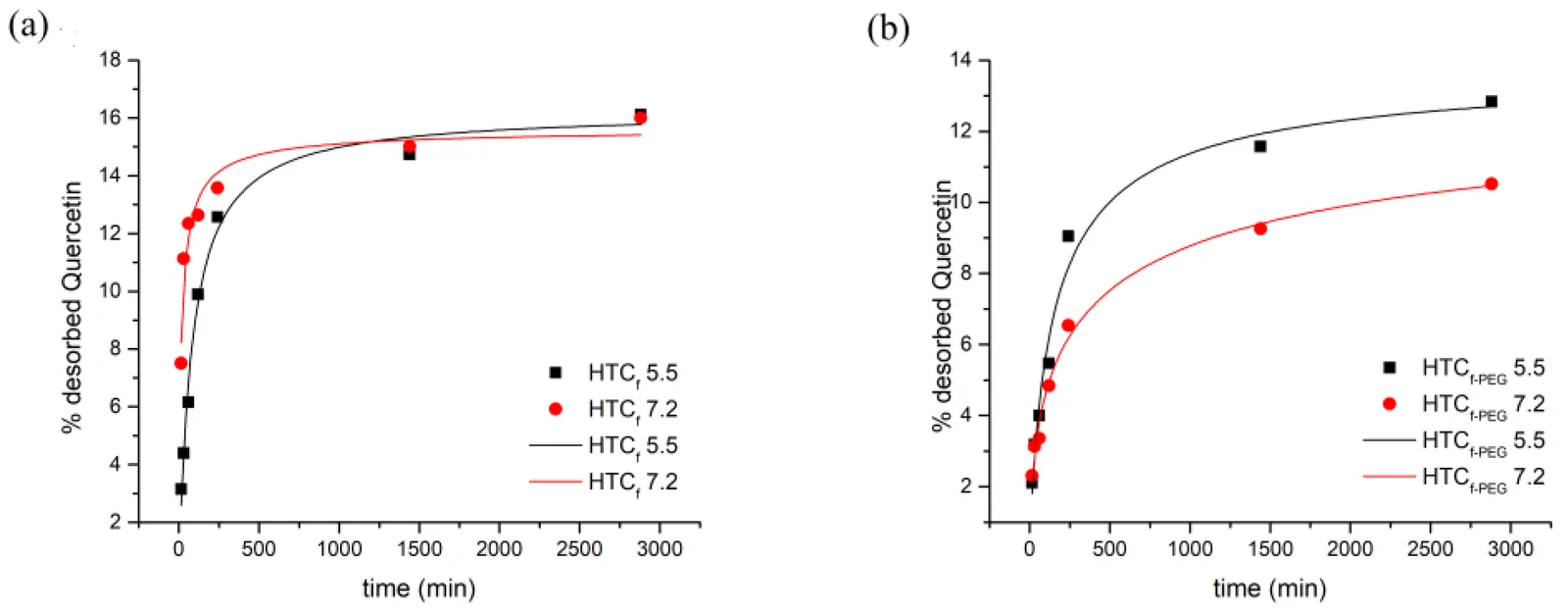
Desorption curves for (**a**) HTC_f_ and (**b**) HTC_f-PEG_ samples at pH values of 5.5 and 7.2.

Desorption curves of quercetin from the investigated samples at different pH values are shown in Figure 7. Desorption experiments were performed for 48 h with shaking. For the HTC_f_ sample (Figure 7a), the results showed that the desorption plateau was reached after 24 h at both pH values. Between 24 h and 48 h, the desorption system is at equilibrium. The desorption curve for the HTC_f-PEG_ sample (Figure 7b) did not reach a plateau after 48 h at either pH, indicating stronger quercetin binding and slower desorption. The maximum desorption achieved after 48 h is 16% for HTC_f_ and about 12% for HTC_f-PEG_.

**Figure 8:**
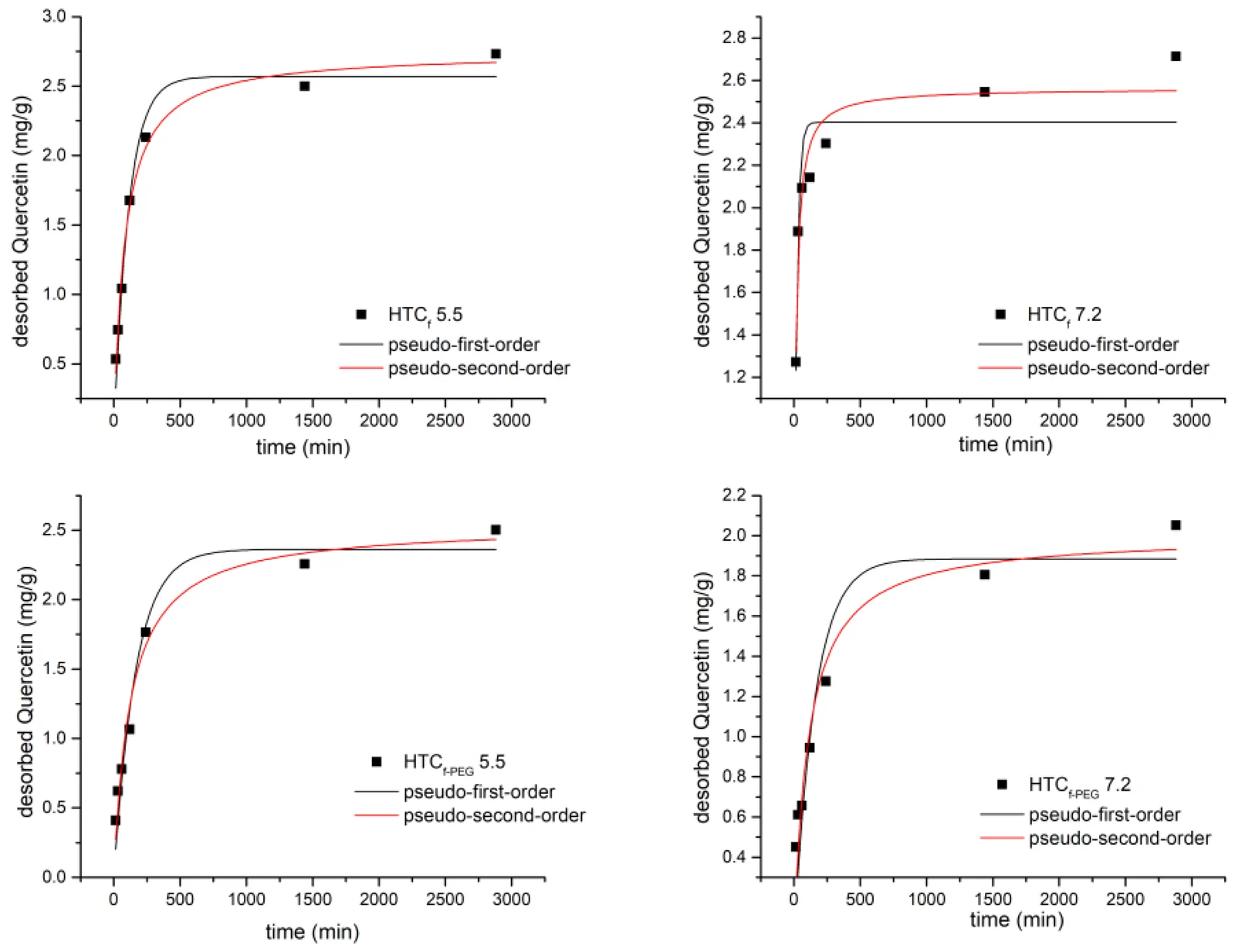
Pseudo-first- and pseudo-second-order kinetics fit for HTC_f_ and HTC_f-PEG_ samples at pH 5.5 and 7.2.

To determine the desorption rate, we fitted the results to pseudo-first- and pseudo-second-order kinetics. Figure 8 shows the fitting graphs, and Table 2 presents the kinetic parameters. The correlation coefficient (R^2^) indicates that the desorption curves for all cases best fit pseudo-second-order kinetics, suggesting that the desorption rate depends on the strength of the bond between quercetin and surface species present on both samples. The fitted equilibrium amounts of desorbed quercetin for HTC_f_ at pH 5.5 and 7.2 are similar, around 2.5 mg/g. For HTC_f-PEG_ at pH 5.5, the fitted equilibrium amount is around 2.3 mg/g, while at pH 7.2, this value is around 1.9 mg/g. The pseudo-second-order rate constant k_2_ (Table 3) for HTC_f-PEG_ is 8–9 times lower compared to HTC_f_ at pH 7.2, which confirms that the functionalization with PEG significantly decreases the quercetin desorption rate compared to the unmodified material.

This decrease may be attributed to PEG becoming a less effective stabilizing agent in acidic solution, as excess protons compete for the same bonding sites that PEG would normally use to interact with quercetin [35]. This competition disrupts PEG–quercetin interactions and consequently facilitates more efficient desorption.

## 4. Conclusions

This investigation shows that PEGylation enables fructose-derived hydrothermal carbon (HTC_f_) to serve as an effective, biocompatible quercetin carrier. Hydrothermal carbonization at 160 °C provides a green, cost-effective synthesis route, yielding carbon microparticles with naturally occurring oxygen-containing functional groups. Although elevated temperatures produced some morphological discrepancies, including irregularly shaped agglomerates, these particles retained sufficient structural integrity and surface chemistry to enable successful covalent functionalization with polyethylene glycol (PEG), as confirmed by FT-IR, XPS, and zeta potential analyses. PEG modification proved crucial for improving material performance, resulting in a 14% increase in quercetin binding capacity. Quercetin desorption analysis showed that, for both samples, desorption curves best fit a pseudo-second-order kinetic model, with the desorption rate constant for the modified sample significantly lower than that for the unmodified sample. This indicates that PEG modification facilitated quercetin bonding in a manner that allowed slow desorption under physiological conditions. Furthermore, the lack of significant cytotoxicity in both non-malignant *MRC-5* and malignant *HeLa* cells confirms the safety of these materials for biological applications. By addressing quercetin’s pharmacokinetic challenges, such as poor bioavailability and low solubility, this study shows that even under elevated temperatures that produce irregular morphologies, fructose-derived HTC_f-PEG_ remains a robust, sustainable, and efficient drug-delivery platform. PEG’s ability to form hydrogen bonds with quercetin increases its solubility and limits crystallization or aggregation, and in this more stabilized molecular state quercetin is assumed to be more readily available for adsorption onto carbon surfaces.

## Figures and Tables

**Table 1:** XPS peak parameters: bond type, binding energy, and atomic percentage.

	Bond Type	Binding Energy (eV)	at%
O1s	OH	532.12	49.26
	COOH	533.92	26.84
	C-O	529.42	23.90
C1s	C=C, C-C, C-H	284.82	49.03
	C-O	286.62	26.92
	C=O	287.72	16.26
	COOH	289.22	7.79

**Table 2:** Zeta potential of carbon materials.

Carbon Material	Zeta (mV)
HTC_f_	−26.4 ± 1.8
HTC_f-PEG_	−16.4 ± 2.6

**Table 3:** Obtained parameters of pseudo-first-order and pseudo-second-order kinetics.

Samples	Pseudo-First-Order		Pseudo-Second-Order	
	R^2^	k_1_, min^−1^	R^2^	k_2_, g mg/min
HTC_f_ 5.5	0.9696	0.0090 ± 0.0011	0.9874	0.0124 ± 0.0013
HTC_f_ 7.2	0.7973	0.0480 ± 0.0095	0.9218	0.0714 ± 0.0120
HTC_f-PEG_ 5.5	0.9552	0.0060 ± 0.0001	0.9741	0.0080 ± 0.0012
HTC_f-PEG_ 7.2	0.8753	0.0063 ± 0.0015	0.9388	0.0088 ± 0.0020

## Data Availability

The dataset is available on request from the authors.

## References

[1] Lachos-Perez D., Torres-Mayanga P.C., Abaide E.R., Zabot G.L., De Castilhos F. (2022). Hydrothermal carbonization and Liquefaction: Differences, progress, challenges, and opportunities. Bioresour. Technol..

[2] Nicolae S.A., Au H., Modugno P., Luo H., Szego A.E., Qiao M., Li L., Yin W., Heeres H.J., Berge N. (2020). Recent advances in hydrothermal carbonisation: From tailored carbon materials and biochemicals to applications and bioenergy. Green Chem..

[3] Bracanović I., Krstić A., Korićanac G., Žakula J., Mirković M., Marković M., Kalijadis A. (2026). Synthesis and functionalization of glucose and fructose derived hydrothermal carbons—Potential application as quercetin carriers. Appl. Surf. Sci..

[4] Krstić A., Lolić A., Mirković M., Kovač J., Arsić T.M., Babić B., Kalijadis A. (2022). Synthesis of nitrogen doped and nitrogen and sulfur co-doped carbon cryogels and their application for pharmaceuticals removal from water. J. Environ. Chem. Eng..

[5] Rafiee Z., Omidi S. (2021). Modification of carbon-based nanomaterials by polyglycerol: Recent advances and applications. RSC Adv..

[6] Cavali M., Dresch A.P., Belli I.M., Libardi Junior N., Woiciechowski A.L., Soares S.R., de Castilhos Junior A.B. (2025). Hydrothermal carbonization—A critical overview of its environmental and economic sustainability. J. Clean. Prod..

[7] Veerapandian M., Ramasundaram S., Jerome P., Chellasamy G., Govindaraju S., Yun K., Oh T.H. (2023). Drug Delivery Application of Functional Nanomaterials Synthesized Using Natural Sources. J. Funct. Biomater..

[8] Sangjan A., Boonsith S., Sansanaphongpricha K., Thinbanmai T., Ratchahat S., Laosiripojana N., Wu K.C.W., Shin H.S., Sakdaronnarong C. (2022). Facile preparation of aqueous-soluble fluorescent polyethylene glycol functionalized carbon dots from palm waste by one-pot hydrothermal carbonization for colon cancer nanotheranostics. Sci. Rep..

[9] Croitoru A.M., Karaçelebi Y., Saatcioglu E., Altan E., Ulag S., Aydoğan H.K., Sahin A., Motelica L., Oprea O., Tihauan B.M. (2021). Electrically triggered drug delivery from novel electrospun poly(Lactic acid)/graphene oxide/quercetin fibrous scaffolds for wound dressing applications. Pharmaceutics.

[10] Niinipuu M., Latham K.G., Boily J.F., Bergknut M., Jansson S. (2020). The impact of hydrothermal carbonization on the surface functionalities of wet waste materials for water treatment applications. Environ. Sci. Pollut. Res..

[11] Nazari-Khanamiri F., Ghasemnejad-Berenji M. (2023). Quercetin and Heart Health: From Molecular Pathways to Clinical Findings. J. Food Biochem..

[12] Georgiou N., Kakava M.G., Routsi E.A., Petsas E., Stavridis N., Freris C., Zoupanou N., Moschovou K., Kiriakidi S., Mavromoustakos T. (2023). Quercetin: A Potential Polydynamic Drug. Molecules.

[13] Aggarwal D., Chaudhary M., Mandotra S.K., Tuli H.S., Chauhan R., Joshi N.C., Kaur D., Dufossé L., Chauhan A. (2025). Anti-inflammatory potential of quercetin: From chemistry and mechanistic insight to nanoformulations. Curr. Res. Pharmacol. Drug Discov..

[14] Wang Y., Tao B., Wan Y., Sun Y., Wang L., Sun J., Li C. (2020). Drug delivery based pharmacological enhancement and current insights of quercetin with therapeutic potential against oral diseases. Biomed. Pharmacother..

[15] Faraji S., Nowroozi N., Nouralishahi A., Shabani Shayeh J. (2020). Electrospun poly-caprolactone/graphene oxide/quercetin nanofibrous scaffold for wound dressing: Evaluation of biological and structural properties. Life Sci..

[16] Rezaei Abbas Abad F., Pourmadadi M., Abdouss M. (2024). Development and characterization of a novel pH-Responsive nanocarrier for enhanced quercetin delivery and cytotoxicity in lung cancer. Inorg. Chem. Commun..

[17] Rezaei Abbas Abad F., Pourmadadi M., Abdouss M., Behzadmehr R., Rahdar A., Pandey S. (2023). Chitosan-Carbon nanotube Composite: An approach for controlled release of Quercetin, Modified with carboxymethyl Cellulose, for potential Anti-Cancer therapy. Inorg. Chem. Commun..

[18] Gautam A., Pal K. (2022). Gefitinib conjugated PEG passivated graphene quantum dots incorporated PLA microspheres for targeted anticancer drug delivery. Heliyon.

[19] Shahzad K., Raza M.A., Hussain A., Ko K.-C., Jeong H.-J., Seralathan K.-K., Han S.S., Park S.H. (2025). Synthesis and characterization of self-crosslinked carboxymethyl chitosan-based hydrogel and its composites with gelatin and PEG-GO for drug delivery applications. Int. J. Biol. Macromol..

[20] Peng Z., Ji C., Zhou Y., Zhao T., Leblanc R.M. (2020). Polyethylene glycol (PEG) derived carbon dots: Preparation and applications. Appl. Mater. Today.

[21] Mostafavi E., Zare H. (2022). Carbon-based nanomaterials in gene therapy. OpenNano.

[22] Wang L., Xu X., Chu L., Meng C., Xu L., Wang Y., Jiao Q., Huang T., Zhao Y., Liu X. (2023). PEG-modified carbon-based nanoparticles as tumor-targeted drug delivery system reducing doxorubicin-induced cardiotoxicity. Biomed. Pharmacother..

[23] Ho T.H., Tong H.D., Trinh T.T. (2024). Molecular insights into the interactions between PEG carriers and drug molecules from Celastrus hindsii: A multi-scale simulation study. Sci. Rep..

[24] Skehan P., Storeng R., Scudiero D., Monks A., McMahon J., Vistica D., Warren J.T., Bokesch H., Kenney S., Boyd M.R. (1990). New Colorimetric Cytotoxicity Assay for Anticancer-Drug Screening. JNCI J. Natl. Cancer Inst..

[25] De Sales L., Bernal-Chávez S.A., Campos-Delgado J. (2026). Functionalization of carbon nanospheres with curcumin, polyethylene glycol and folic acid: Potential use as drug carriers. RSC Adv..

[26] Jung D., Zimmermann M., Kruse A. (2018). Hydrothermal Carbonization of Fructose: Growth Mechanism and Kinetic Model, ACS Sustain. Chem. Eng..

[27] Ischia G., Cutillo M., Guella G., Bazzanella N., Cazzanelli M., Orlandi M., Miotello A., Fiori L. (2022). Hydrothermal carbonization of glucose: Secondary char properties, reaction pathways, and kinetics. Chem. Eng. J..

[28] Bagautdinov B., Ohara K., Babaev A.A. (2022). High-energy X-Ray diffraction study of multiwalled carbon nanotubes fabricated by arc discharge plasma process. Carbon.

[29] Ahn J., Pealer C., Kim S. (2024). Analysis of Carbon Dots Synthesized at Different Hydrothermal Carbonization Temperatures and Their Ferric Ion Detection Performances. Phys. Status Solidi B Basic Res..

[30] Xu Z., Wang J., Guo Z., Xie F., Liu H., Yadegari H., Tebyetekerwa M., Ryan M.P., Hu Y.S., Titirici M.M. (2022). The Role of Hydrothermal Carbonization in Sustainable Sodium-Ion Battery Anodes. Adv. Energy Mater..

[31] Wang L., Wang W., Wang Y., Tao W., Hou T., Cai D., Liu L., Liu C., Jiang K., Lin J. (2024). The Graphene Quantum Dots Gated Nanoplatform for Photothermal-Enhanced Synergetic Tumor Therapy. Molecules.

[32] Vallorz E.L., Encinas-Basurto D., Schnellmann R.G., Mansour H.M. (2022). Design, Development, Physicochemical Characterization, and In Vitro Drug Release of Formoterol PEGylated PLGA Polymeric Nanoparticles. Pharmaceutics.

[33] Iqbal H.M.N., Keshavarz T. (2017). The challenge of biocompatibility evaluation of biocomposites. Biomedical Composites.

[34] Assad M., Jackson N. (2019). Biocompatibility Evaluation of Orthopedic Biomaterials and Medical Devices: A Review of Safety and Efficacy Models. Encyclopedia of Biomedical Engineering.

[35] Ha D.T., Tong H.D., Tran K.-Q., Trinh T.T. (2024). The role of chemical functional groups in dewaterability of hydrochar: A molecular simulation study. J. Mol. Liq..

